# Anti-RA33 antibodies are present in a subset of patients with immune checkpoint inhibitor-induced inflammatory arthritis

**DOI:** 10.1136/rmdopen-2022-002511

**Published:** 2022-09-12

**Authors:** Laura C Cappelli, Clifton O Bingham, Patrick M Forde, Valsamo Anagnostou, Julie Brahmer, Evan J Lipson, Jennifer Mammen, Megan Schollenberger, Ami A Shah, Erika Darrah

**Affiliations:** 1 Division of Rheumatology, Johns Hopkins School of Medicine, Baltimore, Maryland, USA; 2 Department of Oncology, Johns Hopkins School of Medicine, Baltimore, Maryland, USA; 3 Division of Endocrinology, Johns Hopkins School of Medicine, Baltimore, Maryland, USA

**Keywords:** Arthritis, Autoimmunity, Rheumatoid Factor

## Abstract

**Objective:**

Patients with inflammatory arthritis (IA) associated with immune checkpoint inhibitor (ICI) treatment for cancer are typically seronegative for anti-cyclic citrullinated peptide (CCP) antibodies and rheumatoid factor, but little is known about the presence of other autoantibodies in this patient population. We investigated the prevalence and characteristics of anti-RA33 antibodies in patients with ICI-induced IA.

**Methods:**

Anti-RA33 ELISAs were performed on sera from four groups of patients: 79 with ICI-induced IA, 52 with rheumatoid arthritis (RA), 35 treated with ICIs without IA during follow-up and 50 healthy controls. Anti-RA33 positivity and level, clinical and demographic data were compared across groups.

**Results:**

Anti-RA33 antibodies were found in 9/79 (11.4%) patients with ICI-induced IA but in 0/35 patients treated with ICIs who did not develop IA (0%; p=0.04). Of the patients positive for anti-RA33, two had sera available from before ICI treatment; anti-RA33 antibodies were present in both pre-ICI treatments. In patients with RA, 7.7% were positive for anti-RA33 antibodies as were 2% of healthy controls. In ICI-induced IA, anti-RA33 antibodies were associated with anti-CCP antibodies (p=0.001). We found no statistically significant differences in other clinical characteristics in those with and without anti-RA33 antibodies.

**Conclusions:**

Anti-RA33 antibodies are present in a subset of patients with ICI-induced IA, absent in other ICI-treated patients and may be a biomarker for developing IA. Additional studies evaluating serial samples before and after ICI treatment will further establish the temporal relationship of these antibodies to IA development.

**Key messages**

**What is already known on this topic**

- Patients with inflammatory arthritis (IA) due to immune checkpoint inhibitor (ICI) therapy have been primarily seronegative for anti-cyclic citrullinated peptide and rheumatoid factor, but studies have not evaluated the presence of other antibodies associated with early IA, like anti-RA33 antibodies, in this patient population.

**What this study adds**

- The study showed over 11% of patients with ICI-induced IA had anti-RA33 antibodies, while none of the ICI-treated patients without IA had these antibodies; some patients had anti-RA33 antibodies prior to receiving ICI therapy.

**How this study might affect research, practice or policy**

- If validated in future studies, anti-RA33 antibodies could be a biomarker for risk of developing ICI-induced IA.

## Introduction

Immune checkpoint inhibitors (ICIs) improve survival across malignancies.^1^ ICIs target regulatory molecules such as cytotoxic lymphocyte antigen-4, programmed cell death protein 1 and programmed death ligand 1 and can cause excess immune activation leading to immune-related adverse events (irAEs).^2^ IrAEs may resemble rheumatic diseases such as inflammatory arthritis (IA), polymyalgia rheumatica, sicca syndrome and myositis.^3 4^

There are key differences, however, between rheumatic irAEs and traditional autoimmune diseases in clinical characteristics, treatment and biomarkers.^5^ ICI-induced IA is heterogeneous and can persist after cessation of ICI therapy.^6–8^ Imaging has shown synovitis, tenosynovitis, and erosions typical of rheumatoid arthritis (RA), but also enthesitis, enthesophytes, and axial inflammation more characteristic of spondyloarthritis.^9–11^ Greater than 90% of patients with ICI-induced IA lack traditional autoantibodies associated with RA and are negative for HLA-B27, a genetic marker for spondyloarthritis.^8^

Antibodies to heterogeneous nuclear ribonucleoprotein (hnRNP) A2/B1, termed anti-RA33 antibodies, have been described in RA, undifferentiated IA, systemic lupus erythematosus and mixed connective tissue disorder.^12^ These antibodies target a nuclear protein involved in mRNA splicing. A meta-analysis showed that anti-RA33 has high specificity (0.90) for diagnosing RA but low sensitivity (0.33).^13^ RA33 antibodies can be detected in patients with RA who are seronegative for rheumatoid factor (RF) and anti-cyclic citrullinated peptide (CCP) antibodies.^14^ Although the RA33 antigen can be targeted in its native or citrullinated form, antibodies that prefer the native form of hnRNP A2/B1 have been detected in early RA and in patients with low erosion scores on imaging.^15^ The presence of anti-RA33 antibodies targeting the native antigen in early IA and the paucity of antibodies to citrullinated antigens in patients with ICI-induced IA^16^ led us to hypothesise that antibodies to the native RA33 antigen could be present in ICI-induced IA.

## Methods

### Inclusion and exclusion criteria

Four groups of patients were included. All patient biospecimens were collected after approval by the Johns Hopkins institutional review board.

1. Patients with ICI-induced IA (n=79) were included if they had no history of IA or other systemic autoimmune disease before starting ICI therapy, had evidence of IA on examination by a board-certified rheumatologist and had at least one serum sample after diagnosis of ICI-induced IA.
2. Healthy control sera (n=50) was obtained from volunteers who were 18 years of age or older, not pregnant and did not have a history of autoimmune disease, cancer or active HIV, tuberculosis or hepatitis infection.
3. Patients with RA (n=52) were participants in the Johns Hopkins Arthritis Center’s longitudinal database. Patients had rheumatologist-diagnosed RA and were included if they had sera and clinical data available on the same date.
4. Sera from ICI-treated patients who did not develop IA (n=35) were collected through the Johns Hopkins upper aerodigestive malignancy group’s tissue collection protocol. Absence of ICI-induced IA was confirmed with medical record review.

### Anti-RA33 antibody assay

Anti-RA33 IgG antibodies were measured in serum diluted 1:100 using the IMTEC-RA33-Antibodies ELISA kit for the Quantitative Determination of Anti-RA33-IgG Antibodies (#ITC60015, IMETC) according to the manufacturer’s instructions. The cut-off for positivity was set at 3 SD above the mean of the healthy control group.

### Clinical data collection for ICI-induced IA and RA

Patient-reported and physician-reported data were collected in separate longitudinal studies of rheumatic irAEs and RA. Joint counts, patient/physician ratings of disease activity, patient ratings of pain and stiffness, laboratory and imaging findings are recorded at each clinical evaluation.

### Statistical analysis

Descriptive statistics were calculated for demographic and clinical variables. χ^2^ tests were used to compare frequencies of anti-RA33 antibodies. Student’s t-tests or Wilcoxon rank sum tests were used to compare continuous variables in ICI-induced IA patients with and without anti-RA33 antibodies. χ^2^ tests were used to compare categorical variables in those positive and negative for anti-RA33.

## Results

### Demographic features and anti-RA33 frequencies and level

Overall, 216 patients were tested for anti-RA33 IgG antibodies; 79 patients had ICI-induced IA, 50 were healthy controls, 52 had RA and 35 were treated with ICIs without developing IA (table 1). The healthy control participants were the youngest group (p<0.01). The ICI-treated patients without IA had the highest prevalence of former/current smokers and were significantly older than those with IA (p<0.01). The ICI-treated patients with and without IA had a different distribution of tumour types (table 1).

**Table 1:** Characteristics of four included groups

	ICI-IA (N=79) Reference group	ICI without IA (N=35)	RA (N=52)	Healthy controls (N=50)
Age (years), mean±SD	60.4±14.2	68.2±9.1***	57.9±12.6	39.9±10.0***
Female, N (%)	41 (51.9)	13 (37.1)	28 (53.9)	29 (58)
Smoking history, N (%) Never Former Current	43 (58.8) 28 (37.8) 3 (4.5)	3 (8.6)*** 29 (82.8) 3 (8.6)	35 (67.3) 13 (25) 4 (7.7)	34 (68) 15 (30) 1 (2)
Tumour types, N (%)	Melanoma: 27 (34.1) NSCLC: 16 (20.3) GU: 5 (6.3) GI: 11 (13.9) Other: 20 (25.3)	NSCLC: 27 (77%) Mesothelioma: 2 (5.7%) Poorly differentiated: 3 (8.6%) Other: 3 (8.6%)	N/A	N/A
Other irAEs, N (%) None 1 2 or more	35 (44.3) 26 (32.9) 18 (22.8)	21 (60) 12 (34.3) 2 (5.7)	N/A	N/A
Anti-RA33 IgG positive, N (%)	9 (11.4)	0 (0)**	4 (7.7)	1 (2)*
Anti-RA33 level, median (IQR)	0.27 (0, 4.18)	0 (0, 1.69)*	0 (0, 3.89)	0.61 (0, 2.75)

T-test for age. χ^2^ tests for categorical variables. Wilcoxon rank sum test for anti-RA33 level. *p<0.10, **p <0.05 and ***p <0.01.

GI, gastrointestinal; GU, genitourinary; IA, inflammatory arthritis; ICI, immune checkpoint inhibitor; IgG, immunoglobulin; irAEs, immune-related adverse events; NSCLC, non small cell lung cancer; RA, rheumatoid arthritis.

Of the 79 patients with ICI-induced IA, 9 had anti-RA33 antibodies (11.4%, table 1). This was a larger percentage of anti-RA33 positivity than for healthy controls (2%, p=0.05) and ICI-treated patients without IA (0%, p=0.04). There was no statistically significant difference in anti-RA33 positivity between those with ICI-induced IA and RA (7.7%, p=0.48).

**Figure 1:**
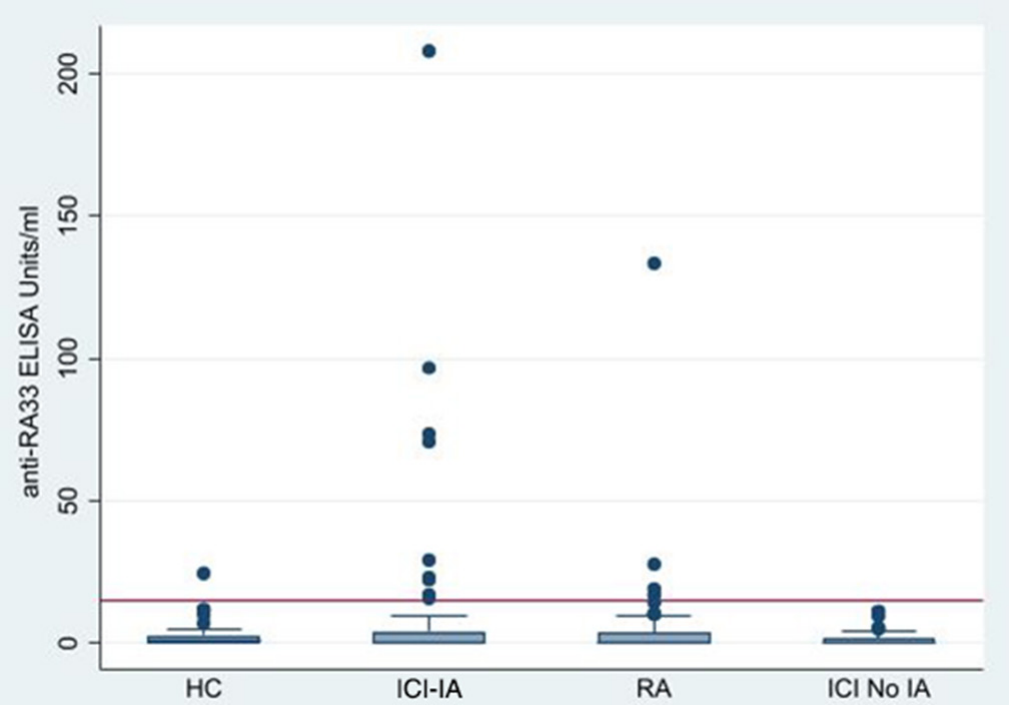
Anti-RA33 levels in immune checkpoint inhibitor-induced Inflammatory arthritis (ICI-IA), healthy controls (HC), rheumatoid arthritis (RA) and ICI-exposed patients without IA (ICI No IA).

Patients with ICI-induced IA had most of the higher level measurements as visualised in figure 1. For those above the cut-off for anti-RA33 positivity, patients with ICI-induced IA had a mean level of 61.8 units and a median level of 29.3 units, while patients with RA had a mean level of 49.3 units and a median level of 23.4 units.

### RA patients and anti-RA33

The mean duration of RA at the time of sample was 9.9 years (SD 10.5). Of the 52 patients, 31 (59.6%) had erosions on imaging, 34 had anti-CCP (65.4%), 30 were positive for RF (57.7%) and 32 had at least one shared epitope allele (61.5%). There were no statistically significant differences in clinical or demographic features between the four patients with anti-RA33 antibodies from the overall population of RA patients tested.

### Clinical features of anti-RA33-positive and anti-RA33-negative patients with ICI-induced IA

ICI-induced IA patients with and without anti-RA33 antibodies did not have significant differences in tumour type or immunotherapy regimen (table 2). People with anti-RA33 antibodies were more likely to have anti-CCP antibodies than anti-RA33-negative individuals (22% vs 1.4%, p=0.001), but had a similar prevalence of RF and antinuclear antibodies (ANA) (p=0.54 and 0.82). The time to develop IA from ICI initiation was similar in both groups, 189 days on average in the anti-RA33-positive group and 209 days in the anti-RA33-negative group (p=0.79). Patients with and without anti-RA33 had similar clinical features at their first rheumatology visit (table 2). Of patients seen in follow-up at least 6 months after ICI cessation, 70.5% still had symptoms of IA or were on medications to control IA symptoms, and there were no differences in persistence of IA by anti-RA33 status (table 2).

**Table 2:** Clinical features of anti-RA33-positive and anti-RA33-negative ICI-IA patients

	Total (N=79)	Anti-RA33 positive (N=9)	Anti-RA33 negative (N=70)	P-value
Age (years), mean±SD	60.4±14.2	62.3±9.5	60.1±14.7	0.66
Female, N (%)	41 (51.9)	7 (77.8)	34 (48.6)	0.10
Type of cancer, N (%) Melanoma NSCLC GU GI Other	27 (34.1) 16 (20.3) 5 (6.3) 11 (13.9) 20 (25.3)	3 (33.3) 2 (22.2) 0 (0) 2 (22.2) 2 (22.2)	24 (34.3) 14 (20) 5 (7.1) 9 (12.9) 18 (25.7)	0.88
ICI regimen, N (%) Anti-PD-1 Anti-PD-L1 Combo PD-1/CTLA-4	52 (65.8) 6 (7.6) 21 (26.6)	8 (88.9) 0 (0) 1 (11.1)	44 (62.9) 6 (8.6) 20 (28.6)	0.29
Time to develop ICI-IA from ICI start in days, median (IQR)	153 (59, 304)	153 (31, 273)	167 (61, 304)	0.79
Prior chemo, N (%)	37 (46.8)	6 (66.7)	31 (44.3)	0.21
Prior radiation, N (%)	27 (34.2)	5 (55.6)	22 (31.4%)	0.15
Additional irAEs, N (%) None 1 2 or more	35 (44.3) 26 (32.9) 18 (22.8)	2 (22.2) 4 (44.4) 3 (33.3)	33 (47.1) 22 (31.4) 15 (21.4)	0.36
CDAI at presentation, median (IQR), N=47	18.1 (11.3, 23)	19.75 (9.25, 33.5)	18.1 (11.3, 23)	0.87
Swollen joint count, median (IQR), N=72	6 (3, 10)	4 (2, 9)	6 (3, 11)	0.73
Patient global median (IQR), N=63	35 (15, 50)	35 (17, 70)	35 (12, 50)	0.53
Stiffness VAS, median (IQR), N=67	50 (33, 75)	70 (45, 75)	50 (30, 75)	0.41
Pain VAS, median (IQR), N=68	50 (21.5, 75)	71 (20, 75)	50 (23, 70)	0.33
Enthesitis, N (%)	20 (25.3)	2 (22.2)	18 (25.7)	0.82
Required corticosteroid, N (%)	65 (82.3)	7 (77.8)	58 (82.9)	0.71
Required csDMARD, N (%)	26 (32.9)	4 (44.4)	22 (31.4)	0.43
Required biologic, N (%)	14 (17.7)	2 (22.2)	12 (17.1)	0.71
RF positive, N (%), N=75	3 (4)	0 (0)	3 (4.5)	0.54
CCP positive, N (%), N=75	3 (4)	2 (22)	1 (1.4)	**0.001**
ANA positive, N (%), N=69	12 (17.4%)	1 (14.2)	11 (17.7)	0.82
ICI persistence (6 months after ICI cessation), N (%), N=51	36 (70.5)	3 (75)	33 (70)	0.84

χ^2^ test for categorical variables. T-test for age. Wilcoxon rank sum for other continuous variables.

ANA, antinuclear antibodies; CCP, anti-cyclic citrullinated peptide; CDAI, Clinical Disease Activity Index; csDMARD, conventional synthetic disease modifying antirheumatic drugs; CTLA-4, cytotoxic lymphocyte antigen-4; GI, gastrointestinal; GU, genitourinary; IA, inflammatory arthritis; ICI, immune checkpoint inhibitor; irAEs, immune-related adverse events; NSCLC, non small cell lung cancer; PD-1, programmed cell death protein 1; PD-L1, programmed death ligand 1; RF, rheumatoid factor; VAS, 100-point Visual Analogue Scale.

### Anti-RA33 pre-ICI treatment

Pre-ICI treatment sera within 1 month of ICI start was available for 15 subjects with ICI-induced IA. Of these 15, 2 had positive anti-RA33 antibodies after ICI treatment, while the rest were negative. Both patients with anti-RA33 antibodies after ICI treatment were also positive for anti-RA33 before treatment, while none of the other 13 were positive in pretreatment sera. Neither patient who was positive for anti-RA33 pretreatment and post treatment was positive for anti-CCP.

## Discussion

This is the first evaluation of autoantibodies beyond ANA, anti-CCP and RF in patients with ICI-induced IA. Anti-RA33 antibodies were detected in 11.4% of patients with ICI-induced IA, whereas none of the ICI-treated controls without IA were positive. For two patients with ICI-induced IA with sera from before ICI treatment, anti-RA33 antibodies were present before ICI exposure. Anti-RA33 antibodies did not correlate with clinical features of ICI-induced IA, except for anti-CCP antibodies. Notably, anti-RA33 antibodies were more prevalent in this cohort of ICI-induced IA than anti-CCP antibodies.

The presence of anti-RA33 antibodies in a subset of patients with ICI-induced IA suggests two main possibilities for their role in pathogenesis. First, anti-RA33 antibodies newly develop as an event in IA initiation post-ICI treatment. Alternatively, anti-RA33 antibodies are present before ICI therapy and are a marker of risk for developing IA. Since antibodies were present pre-ICI in both of the patients who were positive for anti-RA33 and had pre-ICI treatment sera available, it is possible that, as has been reported in patients with anti-CCP antibodies,^17^ there are patients with preclinical IA who manifest clinical IA only after ICI treatment.

There is a question of whether this autoantibody represents an immune response to a tumour antigen since several tumour types overexpress RA33 including glioblastoma, hepatocellular carcinoma and non-small cell lung cancer.^18–20^ Anti-RA33 antibodies were not found in ICI-treated patients without IA, however, suggests that there is some specificity for anti-RA33 antibodies to ICI-induced IA.

While this pilot study revealed anti-RA33 antibodies as potential biomarkers of IA in ICI-treated individuals, there are several limitations to consider, which should be addressed in future studies. One limitation was that ICI-treated controls without IA primarily had lung cancer, while patients with ICI-induced IA had diverse tumours. The predominance of lung cancer in the group of ICI-treated controls likely explains their older age and higher prevalence of smoking. Since RA33 expression has been observed in a range of tumour types, future study of ICI-treated patients with different cancers will be informative. Healthy controls were also significantly younger than other groups which may have affected the rates of autoantibodies detected in these individuals since autoantibodies can accumulate with age. The younger age of healthy controls may have also affected the cut-offs for positivity as this was determined from the healthy control group. Additionally, the study was primarily cross-sectional in nature. It is unclear when anti-RA33 antibodies develop in the course of ICI-induced IA or how long they persist.

Importantly, anti-RA33 antibodies were present in 7.7% of patients with RA in our cohort, which is within the wide range of positivity reported in the literature (6.2%–41%).^14 15 21 22^ While the reasons for the heterogeneity in prevalence between cohorts are unclear, it may reflect differences in ethnic background, disease duration or assay used, since there is no standardised or clinically available anti-RA33 assay. In addition, the rate of positivity in the ICI-IA group was higher than that of the RA group in our study. Other studies interrogating the specificity of anti-RA33 in non-RA disease and healthy controls has found a prevalence of about 3% these other populations, similar to our healthy control prevalence.^14^ Patients with ICI-IA had the highest prevalence of antibodies as compared with all other groups using our assay conditions, which suggests its potential utility as a biomarker in this patient population.

Evaluating immunological factors in ICI-induced IA that are associated with other forms of early IA, such as the presence of anti-RA33 antibodies, may help determine aetiology and define treatment targets. Future studies elucidating the temporal relationship of these antibodies to ICI treatment and validating the presence of anti-RA33 in ICI-induced IA in separate and larger cohorts will help determine the utility of these antibodies in clinical care and their role in pathogenesis.

10.1136/rmdopen-2022-002511.supp1 Supplementary data

## Data Availability

Datasets will be made available upon reasonable request.
